# Supplementary material for: Chromothripsis during telomere crisis is independent of NHEJ, and consistent with a replicative origin
Source: Genome Res. 2019 May;29(5):737–49. doi: 10.1101/gr.240705.118 (PMC6499312; doi:10.1101/gr.240705.118)
Supplement: Supplemental Material [file supp_gr.240705.118_Supplemental_file_1.zip › contigs/annotated_contigs/DB83/contig.2.DB83_length_482_mean_cov_4.3153526971.docx]

**DB83_length_482_mean_cov_4.3153526971**

```
CTCCCCTCC|CCCCCCAACCCGCCTCCCACCCCATATCCGCCCCCCCCCCCCCTCACCCCCACCTCCCTCCCACCCCCCCTCTGCCTCC
          >chr17:16310844-16311259 - E=2e-220 p=0e+00
TCCACCCCTCACCCCACTACCCCTCACCCCCACCTCCCTCCCACCCTCACCTCTGCCTCCTCCACCCCTCATCCCCCGACCCCTCACCC

TCATCTGCCTCACCCCTCCTCCGGCTCCTCCACCCCTCACCCCCCCACCCCTCACCCCCATCTCCCTCCCACCCTTGCCTCTGCCTCCC

CTACCCCTCACTTCGCTACTTTCTTCTCATCCCACCTCAGCCACGCCTCCCACCCTCGCCTCTCCACGCCCTTCCATCCCACTTCAGCC

GCGCCTCCCACCCCTCACACCCCGACCTGCCTCAGCCGCGCCCCCTACCCCGTCCCTCGGGTAGTAGGC|CCCCCCCCGGTTCCGCCCA

GCCCCCCCCCCCCCCCCCCCTCCCCCCCCCC|CCCCCCCC >chr11:61158724-61158755 - E=5e-04
```
